# Wild Type RTA and Less Toxic Variants Have Distinct Requirements for Png1 for Their Depurination Activity and Toxicity in *Saccharomyces cerevisiae*

**DOI:** 10.1371/journal.pone.0113719

**Published:** 2014-12-01

**Authors:** Qing Yan, Xiao-Ping Li, Nilgun E. Tumer

**Affiliations:** Department of Plant Biology and Pathology, School of Environmental and Biological Sciences, Rutgers University, New Brunswick, New Jersey, United States of America; Purdue University, United States of America

## Abstract

Ricin A chain (RTA) undergoes retrograde trafficking and is postulated to use components of the endoplasmic reticulum (ER) associated degradation (ERAD) pathway to enter the cytosol to depurinate ribosomes. However, it is not known how RTA evades degradation by the proteasome after entry into the cytosol. We observed two distinct trafficking patterns among the precursor forms of wild type RTA and nontoxic variants tagged with enhanced green fluorescent protein (EGFP) at their C-termini in yeast. One group, which included wild type RTA, underwent ER-to-vacuole transport, while another group, which included the G83D variant, formed aggregates in the ER and was not transported to the vacuole. Peptide: *N*-glycanase (Png1), which catalyzes degradation of unfolded glycoproteins in the ERAD pathway affected depurination activity and toxicity of wild type RTA and G83D variant differently. PreG83D variant was deglycosylated by Png1 on the ER membrane, which reduced its depurination activity and toxicity by promoting its degradation. In contrast, wild type preRTA was deglycosylated by the free pool of Png1 in the cytosol, which increased its depurination activity, possibly by preventing its degradation. These results indicate that wild type RTA has a distinct requirement for Png1 compared to the G83D variant and is deglycosylated by Png1 in the cytosol as a possible strategy to avoid degradation by the ERAD pathway to reach the ribosome.

## Introduction

Ricin, from *Ricinus communis*, is a heterodimeric protein, which is composed of an A chain (RTA) and a B chain (RTB). The two subunits play distinct roles in the intoxication process. RTA catalytically removes an adenine from the universally conserved α-sarcin/ricin loop (SRL) of the large 28S ribosomal RNA and inhibits protein synthesis [1]. RTB, a cell-binding galactose-specific lectin, promotes endocytosis of ricin [2]. RTA is extremely toxic; a single molecule can inactivate 1500 ribosomes per minute. Because of its high potency and the lack of antidotes, ricin has been used as a bioterrorist weapon and remains a threat worldwide.

Ricin exerts its toxicity by depurinating ribosomes and inhibiting protein synthesis in the cytoplasm. Ricin undergoes retrograde trafficking to enter the cytosol in mammalian cells [2]. The initial step after endocytosis is the delivery of ricin to the early endosomes. A large amount of ricin in the early endosome is either recycled back to the cell surface or delivered via late endosomes to lysosomes. Only a small portion of ricin follows the retrograde pathway from endosomes to the *trans*-Golgi network and subsequently enters the endoplasmic reticulum (ER) [3]. In the ER, RTA is activated through reductive separation from RTB by the protein disulfide isomerase (PDI) [4]. RTA then enters the cytosol by a process termed dislocation or retrotranslocation [5], [6]. The dislocation of RTA is of particular importance, since this is a critical step for intoxication. Due to the high potency of RTA, very few molecules need to reach the cytosol to inactivate the ribosomes. Accumulated evidence suggests that RTA uses components of the ER-associated degradation (ERAD) pathway to reach the cytosol [7]. Transport of misfolded proteins from the ER lumen to the cytosol by the ERAD pathway is critical for many diseases including Alzheimer's and Parkinson's [8]. Viruses subvert this pathway to complete their replication and to escape the immune response [9].

The ERAD components exploited by RTA during its dislocation have been investigated using enzymatically attenuated variants, RTA_E177D_ and RTA_E177A_, as folded proteins and RTA_Δ_, which has a deletion of five amino acids from the active site, as a folding defective protein [10]. These studies were carried out in *S. cerevisiae*, since yeast ribosomes are less sensitive to RTA than mammalian ribosomes [11] and the basic ERAD machinery is conserved between yeast and mammalian systems [7]. Peptide *N*-glycanase (PNGase; yeast Png1), which assists the proteasome mediated degradation of ERAD substrates by deglycosylating *N*-linked unfolded glycoproteins [12], discriminated between the different forms of RTA [10]. Misfolded RTA_Δ_ and RTL (RTA_Δ_ with a transmembrane domain and cytoplasmic *LEU2* marker) were identified as ERAD substrates that required Png1 for deglycosylation and degradation [13]–[15]. In contrast, yeast expressing the folded form, RTA_E177D_, did not show growth defects in response to *PNG1* deletion, leading to the conclusion that Png1 did not affect the folding competent form of RTA [10].

The precise molecular mechanism by which RTA is degraded and how some of this protein escapes degradation by the proteasome in the cytosol remains unclear. We previously showed that wild type RTA, containing the native 35-residue leader of ricin, undergoes ER-to-vacuole transport in yeast [16]. A previous study using the misfolded version of yeast carboxypeptidase, yscY (CPY*) indicated that vacuole transport plays an important role as an alternative degradation pathway when ERAD capacity is saturated by high concentration of substrates or due to defects in the ERAD pathway [17]. Therefore, ER-to-vacuole transport has the potential to act as a degradation pathway and affect the depurination activity and toxicity of RTA. It may also provide an alternative pathway for RTA to enter the cytosol. We showed that a nonglycosylated mutant of RTA, which had similar catalytic activity as wild type RTA, was delayed in vacuole transport and had reduced toxicity and depurination in yeast, suggesting that vacuole transport is important for the depurination activity and toxicity of wild type RTA [16].

Structural features of RTA critical for trafficking are poorly understood. Site-directed mutagenesis and systematic deletion of amino acids resulted in nontoxic RTA variants, whose mutations are clustered at the putative active site cleft [18]–[20]. Random mutagenesis using hydroxylamine identified a series of nontoxic RTA variants bearing mutations away from the active site [21]. These mutations did not eliminate the depurination activity, but reduced the toxicity of RTA. In the present study, we examined intracellular trafficking of RTA variants with reduced toxicity (G83D, G212E, S215F and P95L/E145K) [21] and wild type RTA to determine how intracellular trafficking affects their depurination activity and toxicity. The precursor forms contained the 35-residue leader from ricin at their N-termini, which allows co-translational import of RTA into the ER and subsequent transport to the vacuole [16]. The mature forms do not have the N-terminal leader and remain in the cytosol [16], allowing the examination of their catalytic activity *in vivo* in the absence of trafficking. We show here that wild type RTA and nontoxic RTA variants are sorted differently by the ER quality control system and have distinct requirements for Png1 after dislocation. Png1 mediated degylcosylation and degradation on the ER membrane contributes to the reduced toxicity of an enzymatically active, but nontoxic RTA variant. In contrast the free pool of Png1 degylcosylates wild type RTA in the cytosol and increases its depurination activity possibly by allowing it to evade degradation by the proteasome. These results indicate that Png1 affects both wild type RTA and the G83D variant, but has differential effects on their depurination activity and toxicity.

## Results

### RTA variants differ in intracellular trafficking

**Figure 1:**
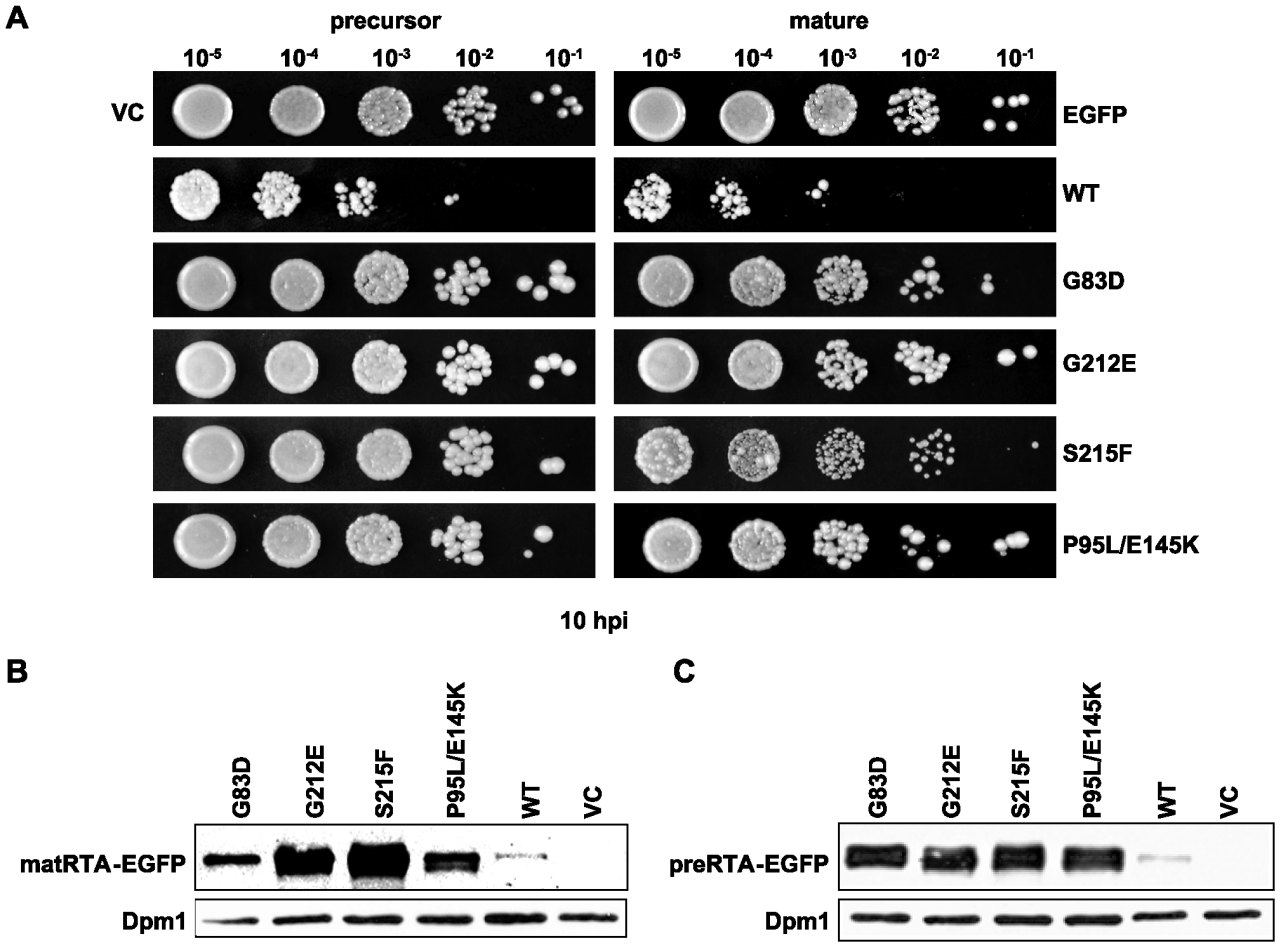
The viability and expression of yeast cells expressing the precursor and mature forms of RTA variants fused to EGFP at their C-termini. (A)Yeast cells were first grown in SD-Leu supplemented with 2% glucose and then transferred to SD-Leu supplemented with 2% galactose. At 10 hpi, a series of ten-fold dilutions were plated on media containing 2% glucose and grown at 30°C for approximately 48 h. The precursor forms are shown on the left and the mature forms are shown on the right. The mutations of RTA are indicated on the right. VC represents empty vector. EGFP represents EGFP open reading frame in the empty vector. Membrane fractions isolated from cells expressing the EGFP tagged mature (B) and precursor (C) forms of wild type RTA and RTA mutants were separated on a 12% SDS-polyacrylamide gel and probed with polyclonal anti-RTA (1∶5000). The blot was reprobed with the ER membrane marker Dpm1p as a loading control.

The precursor and mature forms of RTA variants and wild type RTA were fused to the EGFP tag at their C-termini and expressed in *S. cerevisiae* (W303) under the control of the *GAL1* promoter. The viability assay (Figure 1A) showed that the precursor and the mature forms of RTA variants fused to EGFP were nontoxic, while the precursor and mature forms of wild type RTA were toxic. These results agreed with the previous viability results with the untagged RTA variants [21]. Expression of the mature (Figure 1B) and precursor forms of RTA (Figure 1C) was detected in yeast, and as previously described [22] the level of expression correlated inversely with the viability. A lower level of protein expression was detected if the RTA variant was more toxic. The lowest level of expression was observed with the wild type RTA due to extensive cell death (Figure 1B).

**Figure 2:**
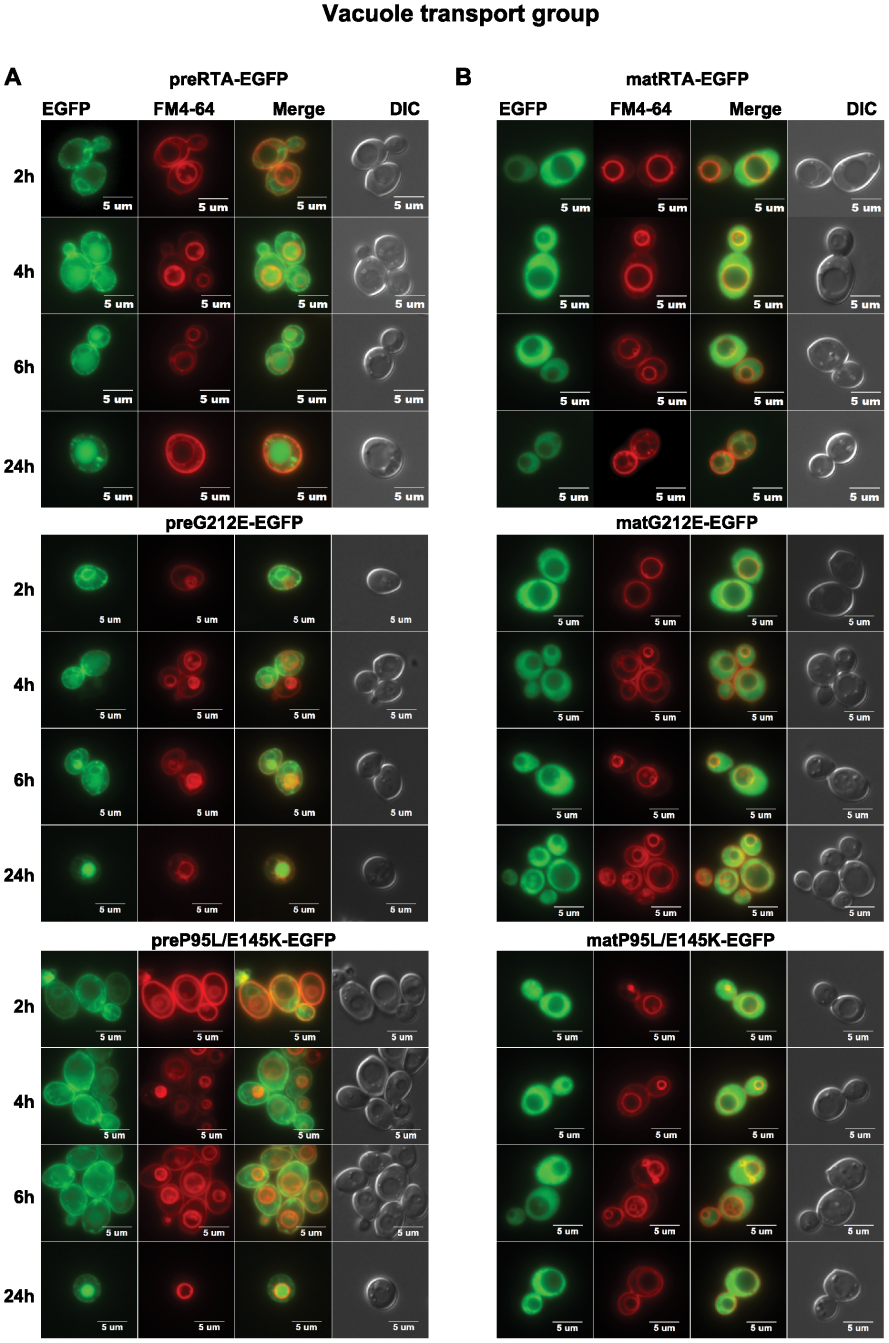
The trafficking of wild type preRTA-EGFP, preG212E-EGFP and preP95L/E145K-EGFP in yeast. Localization of the precursor (A) and mature forms (B) of wild type RTA-EGFP, G212E-EGFP and P95L/E145K-EGFP in yeast (W303) was analyzed at 2, 4, 6 and 24 hpi with an Olympus BX41 epifluorescence microscope. Yeast cells (W303) were treated with FM4-64 to stain the vacuole. Merged images show localization of each protein relative to the vacuole.

The EGFP tag did not affect the sorting and the activity of wild type preRTA as shown previously [16]. Wild type preRTA-EGFP was localized to the ER at 2 hpi, and accumulated in the vacuole at 4 hpi (Figure 2A). At 24 hpi, 100% of the cells showed colocalization of preRTA-EGFP with the vacuole marker FM4-64 [16]. Similarly, preG212E-EGFP and preP95L/E145K-EGFP were both transported to the vacuole as wild type. The preG212E-EGFP colocalized with the ER at 2 hpi, and with the vacuole at 4 hpi and thereafter as wild type preRTA-EGFP. However, the transport of preP95L/E145K-EGFP to the vacuole was delayed by 2 hours. As shown in Figure 2B, the wild-type mature RTA, which did not have the 35-residue leader, was localized in the cytosol after synthesis [16]. Similarly, matG212E-EGFP and matP95L/E145K-EGFP remained in the cytosol.

**Figure 3:**
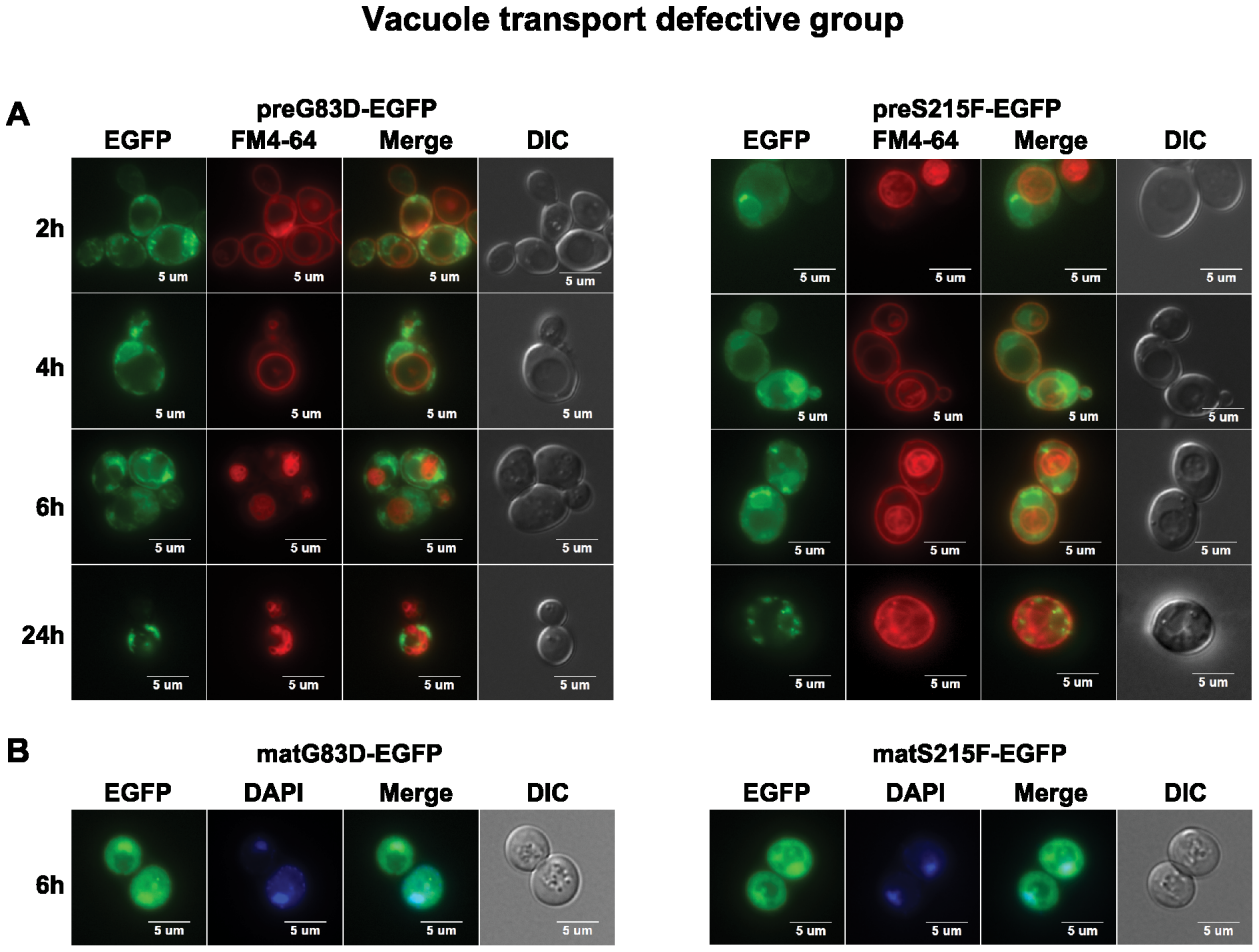
The trafficking of preG83D-EGFP and preS215F-EGFP in yeast. (A) Localization of the precursor forms of G83D-EGFP and S215F-EGFP in yeast (W303) was analyzed at 2, 4, 6 and 24 hpi with an Olympus BX41 fluorescence microscope. Yeast cells were treated with FM4-64 to stain the vacuole. Merged images show that preG83D-EGFP and preS215F-EGFP did not co-localize with the vacuole. (B) The images of yeast cells (W303) harboring the mature forms of G83D-EGFP and S215F-EGFP were taken at 6 hpi. The nuclei were stained with Hoechst 33342. The merged images show co-localization of the matG83D-EGFP and matS215F-EGFP with the nuclei.

The preG83D-EGFP and preS215F-EGFP were not transported to the vacuole even at 24 hpi (Figure 3A). They were retained in the ER and formed large punctate structures on the ER and cell periphery, suggesting that G83D and S215F mutations caused aggregation, which led to transport to the cell periphery from the ER instead of to the vacuole. Besides cytosolic localization, matG83D-EGFP and matS215F-EGFP co-localized with the nuclei, which was shown by staining with Hoechst 33342 (Figure 3B). The nuclear localization implicated the destruction of these proteins by the proteasome, which resides in the nucleus in yeast [23].

### RTA variants differ in depurination activity

**Figure 4:**
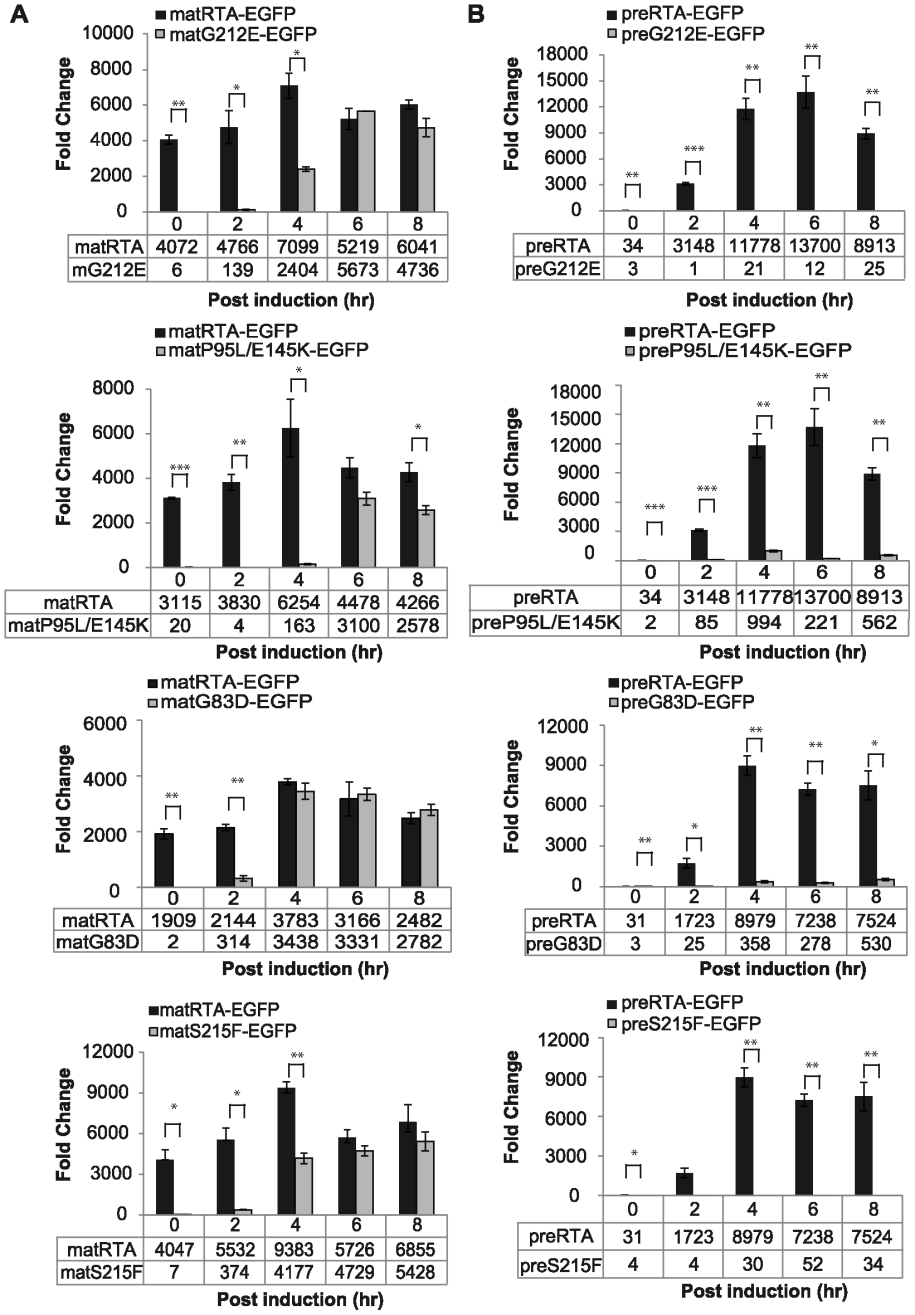
Ribosome depurination of mature (A) and precursor (B) forms of EGFP-tagged RTA variants compared with wild type RTA-EGFP *in vivo*. Ribosomes were isolated from yeast (W303) expressing wild type RTA or each variant at 0, 2, 4, 6 and 8 hpi and depurination was analyzed by qRT-PCR. Two pairs of primers designed to amplify the target amplicon (depurinated SRL) and the reference amplicon (25S rRNA) were used. The data was analyzed by the comparative ΔC_T_ method (ΔΔC_T_). The y-axis indicates the fold change in depurination in yeast harboring the precursor and mature forms of wild type RTA or each variant relative to yeast harboring the empty vector. The tables below each figure show the level of depurination. The data are shown as mean and standard deviation calculated from three replicates. Differences in mean depurination by the RTA variants relative to matRTA (A) or preRTA (B) at each time point were analyzed using two sided, two-sample t tests (*P<0.05, **P<0.01, ***P<0.001).

Ribosome depurination of EGFP tagged precursor and mature forms of RTA variants *in vivo* was analyzed using quantitative RT-PCR [24]. Since the mature forms of RTA remained in the cytosol, they provided a measure of the enzymatic activity *in vivo*, while the depurination of the precursor forms were affected by both trafficking to the cytosol and enzymatic activity. The depurination time-course provided a more sensitive measure of the dislocation of RTA and its activity on yeast ribosomes than the end point cell viability. As shown in Figure 4A, wild type matRTA-EGFP depurinated ribosomes at 0 hpi and the depurination reached a peak at 4 hpi. The vacuole transport competent matG212E-EGFP and matP95L/E145K-EGFP had delayed depurination and the levels were significantly lower than wild type at 0, 2 and 4 hpi. The depurination level of matG212E-EGFP was similar to the wild type at 6 and 8 hpi, while matP95L/E145K-EGFP showed lower depurination than wild type at 8 hpi. These results suggested that vacuole transport competent matRTA-EGFP variants were less active than wild type matRTA-EGFP.

To assess the effects of these mutations on the dislocation of RTA, the *in vivo* depurination level of the precursor forms was analyzed (Figure 4B). Depurination of preRTA-EGFP was detected at 2 hpi, which was delayed compared with matRTA-EGFP, suggesting that ER-to-cytosol dislocation was not a rapid process. The depurination of preG212E-EGFP was negligible throughout the time course. The depurination of preP95L/E145K-EGFP was 12-fold less than preRTA-EGFP at 4 hpi.

The subset of RTA mutants that formed aggregates on the ER membrane showed *in vivo* depurination activity. The matG83D-EGFP was the most active among all the RTA mutants tested. The depurination of matG83D-EGFP at 2 hpi was significantly (7-fold) less than wild type matRTA-EGFP and was similar to wild type at 4 hpi and thereafter (Figure 4A). The depurination of preG83D-EGFP could be detected at 4 hpi and the level was significantly (25-fold) less than preRTA-EGFP (Figure 4B), indicating that the defect in trafficking of preG83D-EGFP caused a large decrease in its depurination. The matS215F-EGFP depurinated ribosomes significantly (15-fold) less than matRTA-EGFP at 2 hpi (Figure 4A). At 4 hpi, the depurination increased, but was still significantly (2-fold) less than matRTA-EGFP. However, the depurination of preS215F-EGFP was significantly reduced compared to wild type (Figure 4B). These results suggested that preG83D-EGFP and preS215F-EGFP were either reduced in transport to the cytosol or were degraded after entry to the cytosol.

### RTA variants are processed differently

**Figure 5:**
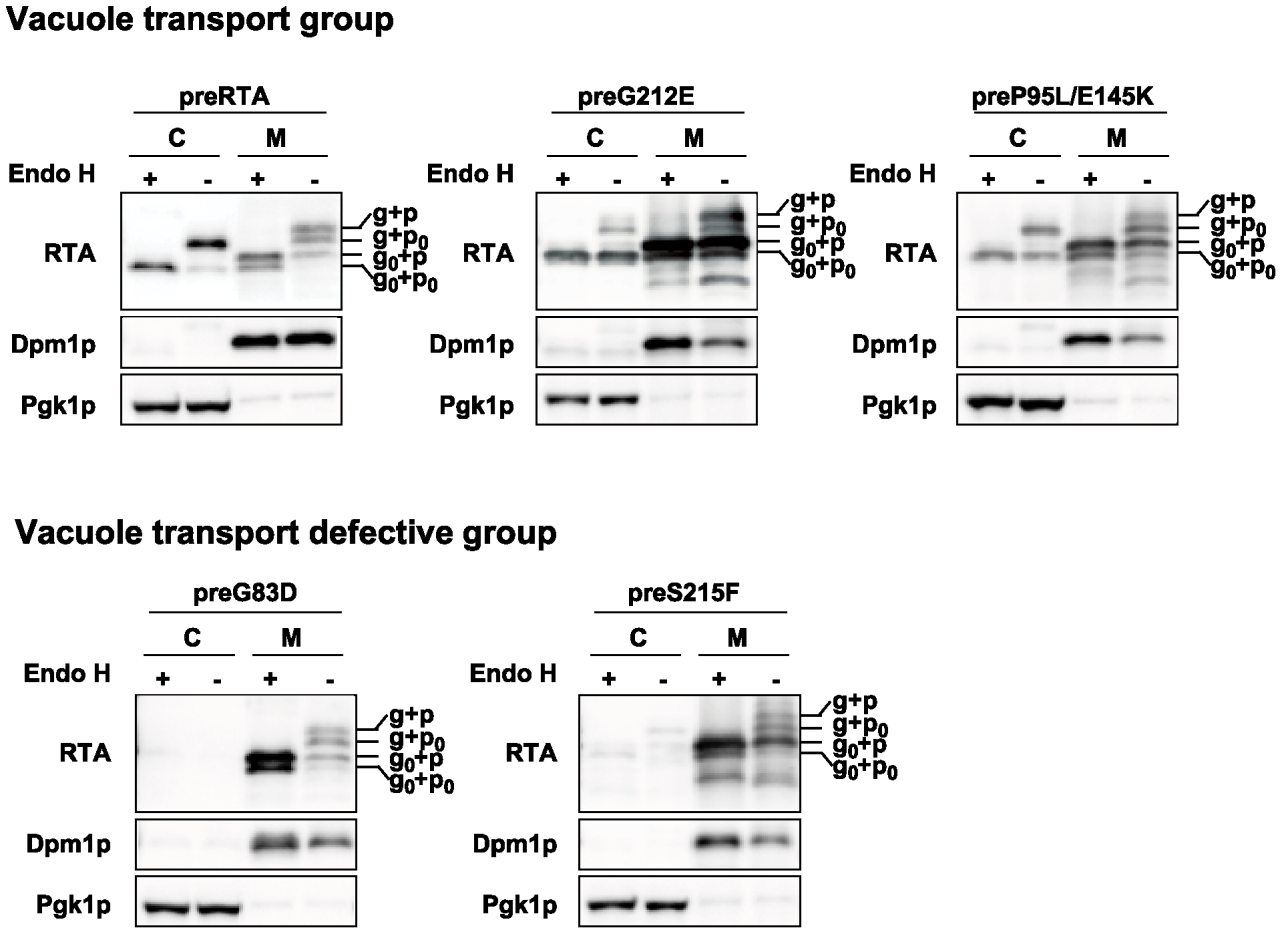
Expression of the wild type RTA and RTA variants. Membrane (M) and cytosol (C) fractions isolated from yeast (W303) at 6 hpi were treated with (+) or without (-) Endo H. The proteins (5 µg) were separated on a 10% SDS-polyacrylamide gel and probed with monoclonal anti-RTA (1∶5000). The blot was reprobed with the ER membrane marker Dpm1p and cytosolic marker Pgk1p as loading controls.

Immunoblot analysis was performed with both membrane and cytosol fraction of yeast (W303) expressing wild type RTA and each nontoxic RTA mutant without the EGFP tag to better resolve the migrating species on SDS-PAGE. The proteins were either treated with (+) or without (-) endoglycosidase H (Endo H) to cleave the mannose rich *N*-linked oligosaccharides. The preRTA migrated as three species in the membrane fraction (Figure 5). The differences in the migration were due to the nine-amino acid propeptide (p) and *N*-glycosylation (g). The top two slower migrating bands represented the glycosylated form with (g+p) or without the nine-amino acid propeptide (g+p_0_). They migrated faster and corresponded to the top and bottom bands respectively after Endo H treatment. The fastest migrating form represented the nonglycosylated and propeptide attached form (g_0_+p), since it corresponded to the band of the same mobility after Endo H treatment. The majority of preRTA was in glycosylated form without propeptide (g+p_0_) in the cytosol and was reduced the size after Endo H treatment. The less abundant faster migrating band in the cytosol was the deglycosylated form of RTA (g_0_+p_0_), which did not change in size after Endo H treatment, suggesting that dislocation to the cytosol occurs only with the propeptide cleaved form of RTA. The nontoxic RTA mutant preG212E migrated as four species in the membrane fraction. Besides the g+p, g+p_0_ and g_0_+p forms that were also present in the wild type, it had a major g_0_+p_0_ band, which was not apparent in the membrane fraction of cells expressing wild type preRTA. The g_0_+p_0_ band might be derived from deglycosylation of the g+p_0_ form. In addition, the glycosylated forms were less abundant compared with nonglycosylated forms of preG212E, suggesting that *N*-glycosylation was not efficient for preG212E or deglycosylation of the ER associated preG212E was rapid. The majority of the protein was in g_0_+p_0_ form in the cytosol. Similarly, preP95L/E145K migrated as four bands in the membrane fraction. The preG83D and preS215F that formed punctate structures showed similar migration on SDS-PAGE (Figure 5). They had g+p, g+p_0_, g_0_+p and a minor g_0_+p_0_ form in the membrane fraction. However, they were not detectable in the cytosol.

**Figure 6:**
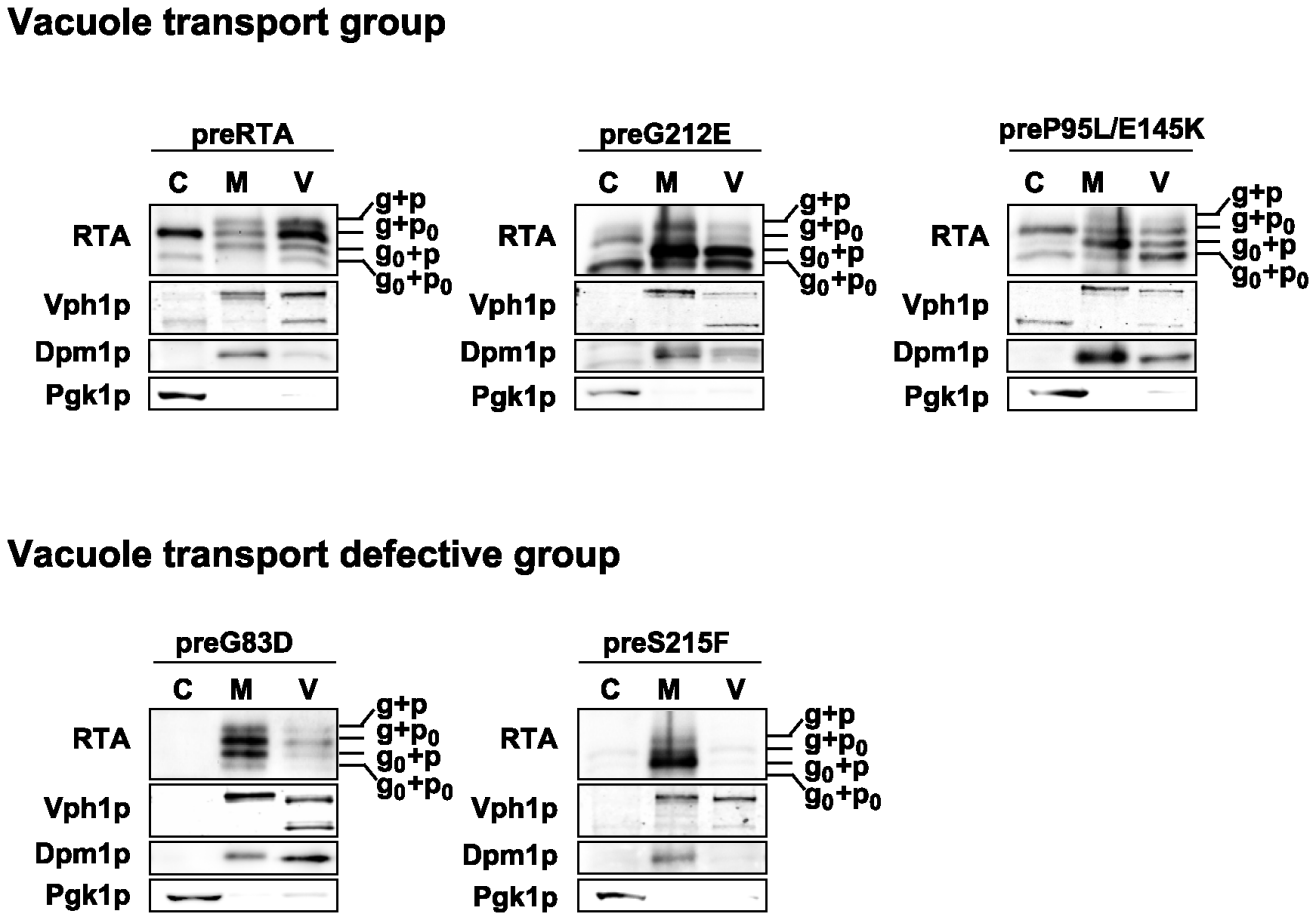
Expression of wild type RTA and RTA variants in vacuole, membrane and cytosol fractions. The vacuole (V), membrane (M) and cytosol (C) fractions were isolated from yeast (W303) at 6 hpi. The proteins (5 µg) were separated on a 10% SDS-polyacrylamide gel and probed with monoclonal anti-RTA (1∶5000). The blot was reprobed with the vacuole membrane marker, Vph1p, the ER membrane marker, Dpm1p and the cytosol marker, Pgk1p.

To determine if RTA variants are transported to the vacuole as wild type preRTA, the vacuole fraction was isolated from yeast expressing preRTA, preG212E and preP95L/E145K, as well as preG83D and preS215F without the EGFP tag at 6 hpi (Figure 6). Immunoblot analysis showed that preRTA, preG212E and preP95L/E145K were present in the vacuole fraction at 6 hpi, whereas preG83D and preS215F were absent (Figure 6), consistent with epifluorescence microscopy analysis (Figures 2A and 3A). Although the vacuole fraction contained some ER membrane contamination based on the loading control, Dpm1, the amount was less than in the membrane fraction and distribution of the different forms of RTA in the vacuole fraction and in the membrane fraction were different, suggesting that the proteins present in the vacuole fraction were not due to the ER contamination. The vacuole marker, Vph1p, encoding the vacuolar H^+^-ATPase was 100 kDa in the membrane fraction, but partially reduced to 75 kDa in the vacuole fraction, due to proteolysis during the vacuole isolation [25]. The preRTA migrated as four species in the vacuole, including g+p, g+p_0_, g_0_+p and g_0_+p_0_. These results suggested that the propeptide attached forms are exclusively sorted to the vacuole from the ER and are processed in the vacuole. The vacuole fractions of yeast expressing preG212E and preP95L/E145K also contained all four migrating species. However, the abundance of different forms was not the same as wild type RTA, suggesting that preG212E and preP95L/E145K are processed differently. Yeast expressing preG83D and preS215F had very low amount of proteins in the vacuole fraction, which could be due to the contamination from the ER membrane. These results demonstrated that preG83D and preS215F are defective in vacuole transport and are only associated with the ER membrane, while preG212E and preP95L/E145K are sorted to the vacuole.

### Png1 affects the toxicity and depurination activity of wild type and variant forms of RTA differently

Previous studies showed that Png1 was responsible for the deglycosylation of folding defective RTA_Δ_ and RTL and assisted in their degradation by the proteasome [10], [14]. Moreover, yeast Png1 could distinguish between the native form and the unfolded form of a glycoprotein [26]. We transformed the precursor form of wild type RTA and nontoxic RTA variants into *png1*Δ and the parental BY4743 strain, to determine if *PNG1* deletion affected their deglycosylation and degradation. The viability, deglycosylation and depurination level of vacuole transport competent preG212E and preP95L/E145K (Figure S1A–C) and vacuole transport defective preS215F (Figure S2A–C) were similar in *png1*Δ and in the parental strain. However, Png1 affected both wild type preRTA and preG83D, but the responses were different.

**Figure 7:**
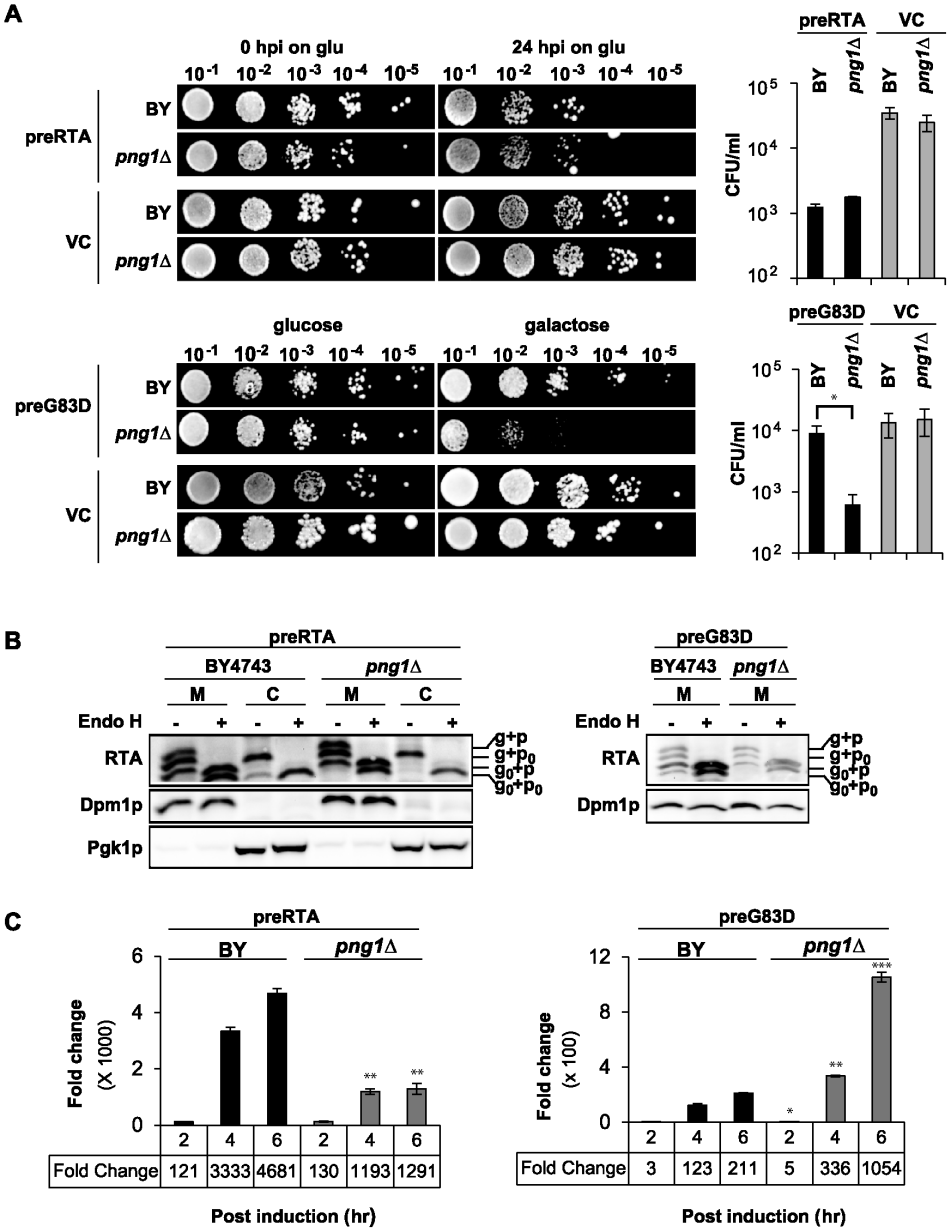
Analysis of preRTA and preG83D in *png1Δ*. (A) The viability of *png1Δ* and isogenic wild type (BY4743) expressing preRTA or preG83D. A series of 10-fold dilutions were spotted either on a glucose plate at 0 and 24 h after galactose induction or on a galactose plate after overnight growth in glucose media. The CFU/ml was calculated based on the analysis of at least three different transformants. Differences in CFU/mL for BY4743 and png1Δ were analyzed using two sided, two-sample t tests (*P<0.05). (B) Immunoblot analysis of membrane and cytosol fractions of *png1*Δ and BY4743 expressing preRTA or preG83D. The proteins (5 µg) were treated with (+) or without (-) Endo H. (C) Ribosome depurination by preRTA or preG83D was analyzed by qRT-PCR after isolation at 2, 4, 6 hpi. Differences in mean depurination by preRTA or preG83D in BY4743 and png1Δ were analyzed using two sided, two-sample t tests (*P<0.05, **P<0.01, ***P<0.001).

The *png1*Δ and wild type (BY4743) expressing preRTA were induced in galactose for 24 h and plated on non-inducing glucose to compare the viability at 24 hpi. Quantification of colony forming units (CFU/ml) indicated that viability of *png1*Δ expressing preRTA was similar to BY4743 expressing preRTA (Figure 7A). The *png1*Δ and BY4743 expressing preG83D were plated directly on galactose, since the difference in viability was not obvious on glucose due to the reduced enzymatic activity of preG83D. BY4743 expressing preG83D was significantly (15-fold) more viable than *png1*Δ expressing preG83D (Figure 7A). These results suggested that Png1 is involved in the degradation of preG83D.

Immunoblot analysis of preG83D and preRTA was performed with both membrane and cytosol fractions treated with (+) and without (-) Endo H treatment to distinguish the glycosylated forms (Figure 7B). The preRTA showed three bands in the membrane fraction, corresponding to g+p, g+p_0_ and g_0_+p forms in BY4743 as in W303 (Figure 5). The g+p_0_ form and the deglycosylated g_0_+p_0_ form were observed in the cytosol, suggesting that the glycosylated RTA was deglycosylated in the cytosol. Similar forms of RTA were observed in the membrane fraction in *png1*Δ as in BY4743. However, the deglycosylated form of RTA was not visible in the cytosol in *png1*Δ. These results suggested that the deglycosylation of preRTA in the cytosol was compromised in the absence of Png1. The preG83D migrated as four bands, corresponding to g+p, g+p_0_, g_0_+p and g_0_+p_0_ forms in the membrane fraction in BY4743. The preG83D could not be detected in the cytosol in BY4743 as in W303 (Figure 5). In *png1*Δ, the deglycosylated form of G83D without the propeptide (g_0_+p_0_) disappeared from the membrane fraction and the g_0_+p form became less abundant, suggesting that Png1 is involved in the deglycosylation of the membrane associated preG83D. These results suggested that Png1 deglycosylates wild type preRTA in the cytosol and preG83D on the ER membrane.

Depurination was quantified using qRT-PCR as a sensitive method to measure the catalytic activity in the cytosol (Figure 7C). The depurination of preRTA increased slowly in *png1*Δ and was significantly (2.8 and 3.6-fold) lower than in BY4743 at 4 and 6 hpi, respectively. The decrease in the depurination level of preRTA in *png1*Δ was not a global effect, since the depurination level of the nonglycosylated preN10Q/N236Q, which is not a substrate for Png1 [14], was not affected in *png1*Δ (Figure S3A–B). The reduced depurination of wild type preRTA in *png1*Δ suggested that Png1 deglycosylates preRTA and increases its depurination activity in the cytosol, rather than promoting its degradation. In contrast, preG83D expressed in *png1*Δ showed significantly (3-fold and 5-fold) higher depurination than in BY4743 at 4 and 6 hpi, respectively (Figure 7C). The increased depurination of preG83D in *png1*Δ suggested that Png1 is involved in degradation of preG83D.

**Figure 8:**
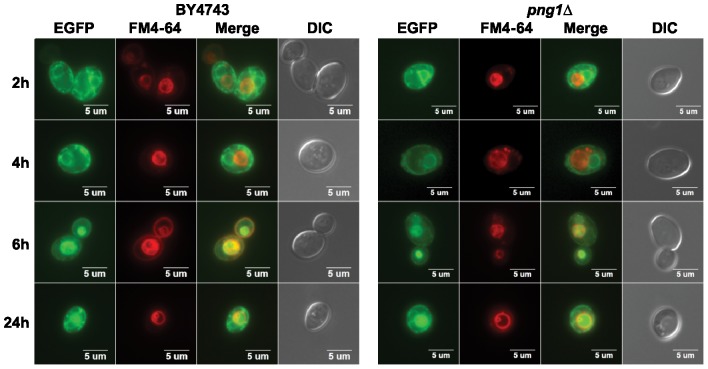
The trafficking of preRTA-EGFP in *png1*Δ. The *png1*Δ and isogenic wild type (BY4743) harboring preRTA-EGFP was visualized at 2, 4, 6 and 24 hpi with an Olympus BX41 fluorescence microscope. Yeast cells were treated with FM4-64 to stain the vacuole. Merged images show localization of preRTA-EGFP relative to the vacuole.

The reduced depurination by wild type preRTA in *png1*Δ suggested that vacuole transport of wild type preRTA might be affected in *png1*Δ. To address this preRTA expressed in *png1*Δ and in the parental BY4743 was analyzed by epifluorescence microscopy (Figure 8). The majority of preRTA-EGFP was localized to the ER at 2 and 4 hpi in BY4743. At 4 hpi, preRTA-EGFP was localized to the vacuole in only 3% (*n* = 100) and at 6 hpi in 34% (*n* = 100) of the cells. The vacuole transport of preRTA occurred at the same time in *png1*Δ. At 4 hpi, 2% (*n* = 100) and at 6 hpi, 37% (*n* = 100) of the cells showed preRTA-EGFP in the vacuole in *png1*Δ. These results suggested that the vacuole transport of preRTA was not affected by the *PNG1* deletion.

To determine if the phenotype of *png1*Δ expressing wild type RTA and preG83D was due to the absence of Png1, we tested if expression of yeast *PNG1* driven by the constitutive *GPD1* promoter would complement the phenotype of *png1*Δ expressing wild type RTA or preG83D. Expression of *GPD1* driven *PNG1* reduced the viability of *png1*Δ expressing preRTA to a similar level as BY4743 expressing preRTA (Figure S4A). Expression of *PNG1* was confirmed by immunoblot analysis with anti-HA (Figure S4B). Expression of *PNG1* in *png1*Δ increased the depurination level of wild type preRTA to a similar level as in BY4743 expressing preRTA (Figure S4C). Expression of *PNG1* restored the viability of *png1*Δ expressing preG83D (Figure S5A). Expression of preG83D and *PNG1* was confirmed by immunoblot analysis using anti-HA (Figure S5B). Expression of *PNG1* reduced depurination in *png1*Δ expressing G83D to a similar level as in BY4743 expressing preG83D (Figure S5C). These results demonstrated that Png1 was responsible for the phenotypes observed in *png1*Δ expressing either wild type preRTA or preG83D.

## Discussion

### Mutations in RTA affect not only catalytic activity, but also intracellular trafficking

In previous studies dislocation of RTA from ER was examined by targeting RTA variants with reduced toxicity to the ER using the heterologous Kar2 signal sequence [10], [13], [14], [27]. Here we used a similar approach to compare the trafficking of a series of catalytically active RTA mutants with reduced toxicity [21] to wild type RTA in yeast. However, in our study we used the native 35-residue leader from ricin, which contains the 26-residue signal peptide for co-translational import of RTA to the ER and the 9-residue propeptide, which targets RTA to the vacuole [16]. Vacuole transport of RTA was not observed in previous studies, possibly because RTA variants have been targeted to the ER using heterologous signal peptides [10], [13], [14], . We compared the trafficking of wild type RTA to RTA variants with point mutations, which reduced the toxicity but did not eliminate the depurination activity of the mature forms *in vivo*. The cytotoxicity, depurination and cellular trafficking of the precursor and mature forms of RTA variants are summarized in Table 1. Based on the trafficking pattern, RTA variants were divided into two major groups. The first is the vacuole transport group, which included wild type preRTA, preG212E and preP95L/E145K. Their expression was detected in the membrane, cytosol and vacuole fraction as wild type RTA, but with an expression pattern different from wild type RTA. The second group included vacuole transport defective RTA variants, preG83D and preS215F, which were retained in the ER and formed large punctate structures on the ER and cell periphery. Their expression was detected only in the membrane fraction and not in the cytosol or in the vacuole. In both groups, the precursor forms had lower depurination activity than the mature forms, indicating that either ER-to-vacuole trafficking or defects in ER-to-vacuole trafficking reduced the depurination activity of RTA. More importantly, mutations outside the active site, which reduced the depurination activity of the mature forms, had different effects on the depurination activity of the precursor forms, indicating that they affected trafficking differently. RTA variants with reduced catalytic activity are often used to study the trafficking pathway exploited by RTA. We show here that these variants may not only have reduced catalytic activity, but they may also follow different intracellular trafficking pathways depending on the particular mutation. Our results also underscore the necessity to examine both the precursor and mature forms of RTA to distinguish between the effects on catalytic activity and on trafficking.

**Table 1:** Toxicity, depurination and trafficking of RTA variants.

	precursor			mature		
	toxicity	depurination	trafficking	toxicity	depurination	trafficking
WT	+	++++	ER-to-vacuole	+	+++++	cytosol
G212E	-	-	ER-to-vacuole	-	+++	cytosol
P95L/E145K	-	+	ER-to-vacuole delayed	-	++	cytosol
G83D	-	+	ER	-	++++	cytosol and nucleus
S215F	-	-	ER	-	+++	cytosol and nucleus

The propeptide attached glycosylated and unglycosylated forms of preRTA were detected exclusively in the vacuole fraction, and not in the cytosol [16], suggesting that they do not go through the ERAD pathway but are transported to the vacuole. In contrast, the propeptide cleaved glycosylated form was detected in the cytosol. These observations suggest that the propeptide of preRTA is an important sorting signal for transport to the vacuole. This is in agreement with our previous study, which showed that deletion of the propeptide delayed vacuole delivery of preRTA in yeast [16]. They are also consistent with the observation that ricin propeptide is processed in the vacuole in plants [28]. The propeptide may influence vacuole transport by facilitating the folding of preRTA as was suggested for CPY [29], [30]. The propeptide cleaved glycosylated form of RTA was the major form observed in the cytosol, suggesting that dislocation to the cytosol occurs after vacuole transport.

Several lines of evidence demonstrated that RTA undergoes anterograde trafficking from the ER to the Golgi [10], [13], [31]. Golgi apparatus is part of the ER quality control machinery and contains anterograde transport and retrograde transport dynamics [32]. Therefore, the delivery of preRTA to the vacuole may be preceded by anterograde transport to the Golgi. Similarly, the canonical ERAD substrate CPY* was also shown to be sorted to two competing pathways within the ER, ERAD and ER exit followed by vacuole delivery. The CPY* mutant carrying the ER exit signal to the vacuole had a faster degradation rate than the CPY* mutant acting only as an ERAD substrate, since the rate of vacuole turnover is higher than the ERAD pathway [33]. The deglycosylated preG212E was more abundant in the vacuole fraction than preP95L/E145K (Figure 6). Moreover, preG212E-EGFP was observed in the vacuole earlier than preP95L/E145K-EGFP (Figure 2A). The faster vacuole delivery of preG212E-EGFP compared to preP95L/E145K-EGFP may explain why preG212E-EGFP depurinated ribosomes at a lower level than preP95L/E145K-EGFP (Figure 4B) even though G212E was more active (Figure 4A). These results imply that ER-to-vacuole transport is a degradation pathway for wild type RTA. However, since vacuole transport is critical for the toxicity of RTA [16], a small amount of the propeptide cleaved glycosylated form of wild type RTA can escape vacuole degradation and can enter the cytosol. A genome-wide RNAi screen against ricin in mammalian cells identified several genes involved in vacuolar protein sorting, which were protective against ricin when knocked down [31]. Similarly COPII components required for anterograde trafficking from the ER were protective against ricin when knocked down, suggesting that preventing anterograde trafficking from the ER prevents delivery of ricin to the ER [31]. Taken together these results suggest that preRTA containing the native 35-residue leader may enter the cytosol from the ER after anterograde trafficking to the vacuole.

### Png1 deglycosylates wild type RTA in the cytosol and contributes to its depurination activity and toxicity

Dislocation of RTA to the cytosol is a critical step in intoxication. The toxin is postulated to hijack the ERAD machinery to enter the cytosol [7]. However, it is not known how wild type RTA evades degradation by the proteasome after entry into the cytosol. The low number of lysines in RTA (only two) has been suggested as a possible strategy to limit proteasome dependent degradation [34]. Folding defective RTA variants were shown to dislocate to the cytosol from the ER through the ERAD-L pathway [10]. A crucial structural element required for substrate recognition in the ERAD pathway is *N*-linked glycans. PNGase catalyzes the deglycosylation of unfolded glycoproteins in the cytosol and assists their degradation by the proteasome [12]. PNGase has stringent structural constraints for substrate deglycosylation. Yeast Png1 prefers high mannose type oligosaccharides [35]. The truncated glycoproteins lacking terminal mannose residues do not act as substrates for Png1 [36]. Png1 could distinguish between the folded and denatured glycoproteins *in vitro* [26]. However, the role of Png1 in ERAD is not clear and the structural determinants for Png1 recognition are not known.

**Figure 9:**
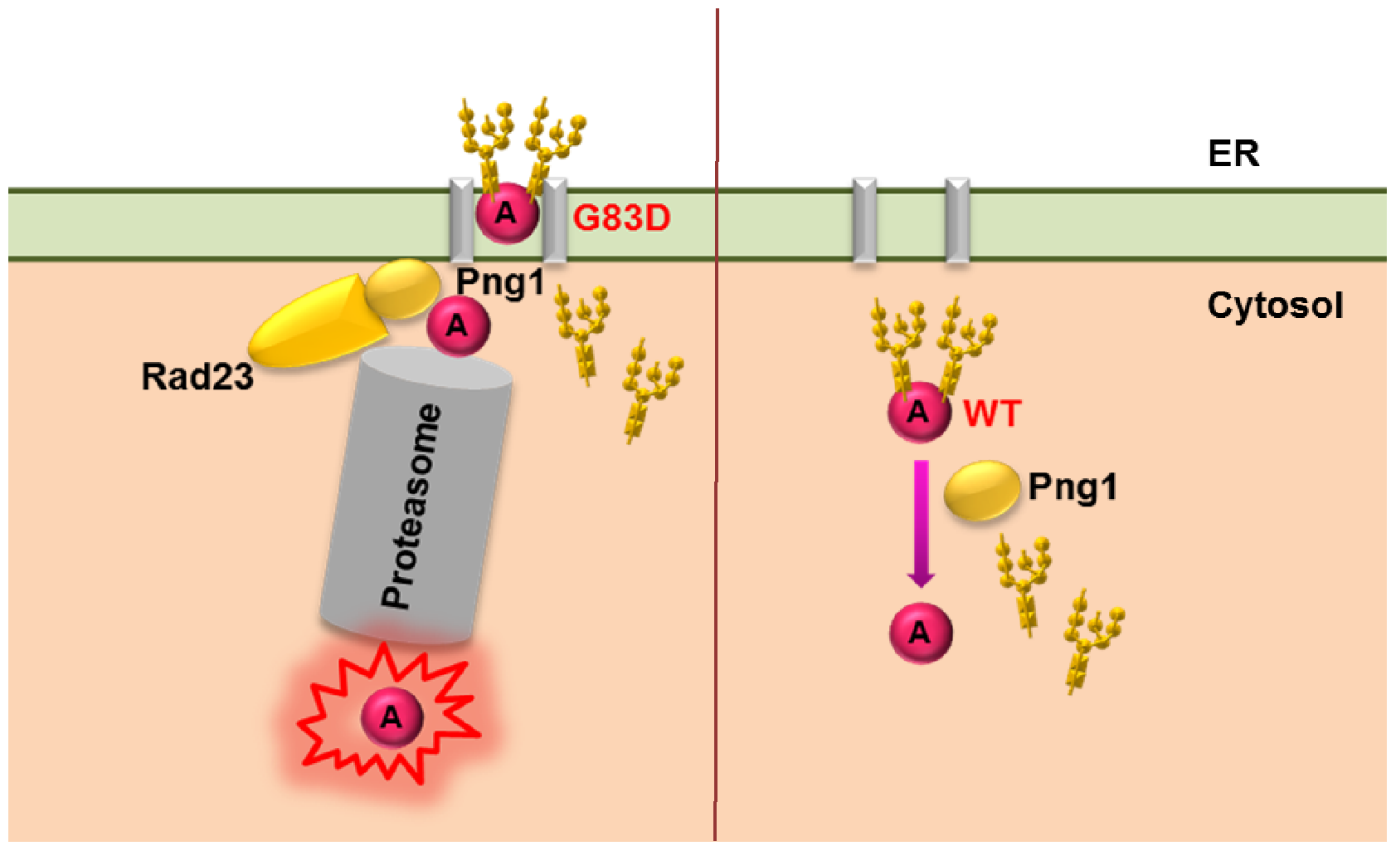
A model describing the involvement of Png1 in the deglycosylation of preG83D and wild type preRTA. The preG83D is deglycosylated by the Png1 on the ER membrane and is targeted to the proteasome for degradation. Wild type preRTA is deglycosylated by the free pool of Png1 in the cytosol, preventing its degradation by the proteasome.

We show here that ribosome depurination by the preG83D variant increased (Figure 7C) and viability decreased (Figure 7A) in response to *PNG1* deletion, suggestive of a decrease in degradation. Since *N*-linked glycans can stabilize folding intermediates, the preG83D variant was likely stabilized in *png1*Δ. However, the protein level corresponding to the preG83D variant did not increase in *png1*Δ (Figure 7B) because preG83D was more toxic in *png1*Δ than in BY4743 (Figure 7A). In contrast, removal of *N-*glycan led to further destabilization of preG83D in BY4743 and hence to its degradation by the ERAD pathway. In both mammalian and yeast systems, PNGase is proposed to exist as a free pool and an ER-associated pool in complex with Rad23/HR23B which functions in releasing the glycan chains from the proteins to meet the conformational constraints in protein degradation by the proteasome [13], [37]. A previous study demonstrated that a specific mutation on Rad23 which abolished its interaction with Png1 could not restore the degradation of a misfolded RTA mutant in *rad23Δ*, indicating that Png1 has to be coupled with Rad23 to degrade misfolded RTA and that free Png1 was not involved in the degradation [13]. Our results indicate that Png1 deglycosylates the preG83D variant on the ER membrane. The preG83D variant may be deglycosylated by the Png1-Rad23 complex [37] and sent to the proteasome linked with Png1 for destruction [38]. These results fit a model where the G83D variant, which forms aggregates, is deglycosylated by ER-associated Png1 on the ER membrane and targeted to the proteasome for degradation as a typical ERAD substrate (Figure 9).

In previous studies, *PNG1* deletion did not affect the growth of cells expressing the folding competent RTA_E177D_ [10], but decreased the viability of cells expressing the folding defective RTA_Δ_, suggesting that the unfolded form, but not the folded form of RTA was degraded by a Png1-dependent pathway [13]. Although the deglycosylated form of folded RTA_E177D_ was detected in the cytosol in yeast, plant and mammalian cells [27], [39], [40], the mechanism was not addressed. We show here that the viability of yeast expressing wild type preRTA did not change appreciably upon *PNG1* deletion (Figure 7A) consistent with previous results [10]. However, depurination by wild type preRTA decreased (Figure 7C), indicating that depurination is a more sensitive assay than viability. Deglycosylation of wild type preRTA was also affected in *png1*Δ (Figure 7B). The reduced depurination by wild type preRTA in *png1*Δ was *N-*glycan dependent, because preRTA lacking the *N*-glycosylation sites (preN10Q/N236Q) showed similar level of depurination in *png1*Δ as in wild type (Figure S3). The deglycosylated RTA has been shown to be more stable than the glycosylated form [13]. Hence reduced depurination by preRTA in *png1*Δ may be due to the higher degradation rate of the glycosylated form of RTA compared with the deglycosylated form.

The C-terminal hydrophobic region is necessary for RTA to get out of the ER [16]. RTA is thought to be recognized as an ERAD-L substrate by interaction of this C-terminal hydrophobic region with ER lipids upon reduction of the holotoxin in the ER [41]. Dislocated RTA is thought to rapidly refold in the cytosol to gain catalytic activity and to escape proteasomal degradation [42]. We previously showed that removal of *N*-glycan does not affect the catalytic activity of RTA [16]. Therefore, deglycosylation of wild type preRTA by Png1 increases depurination not by increasing the catalytic activity of preRTA, but possibly by increasing its stability. The folded wild type RTA may not be deglycosylated by Png1 during exit from the ER because it does not exit the ER through the ERAD pathway as a typical ERAD substrate. It may be deglycosylated by the free Png1 in the cytosol because there is no Png1 present at the ER exit sites, and hence deglycosylation of wild type preRTA by Png1 occurs in the cytosol (Figure 9).

Although preS215F formed aggregates like preG83D and was obviously a misfolded protein, it did not respond to *PNG1* deletion (Figure S2), suggesting that this mutant was not a substrate for Png1. This is consistent with previous results, which showed that not all glycoproteins are degraded by Png1 [13]. The structural selectivity of Png1 and how it deciphers the structural elements in glycoproteins are not well understood. It is possible that preS215F is not recognized by Png1 due to differences in the structure of its oligosaccharide chains. The preG212E and preP95L/E145K closely resemble wild type preRTA in their intracellular trafficking. However, *PNG1* deletion did not affect their depurination activity either. Since these mutants already had very low depurination activity, a further decrease in depurination caused by *PNG1* deletion might not have been detected. The expression pattern of these mutants was similar in *png1*Δ or in the parental strain, and the deglycosylated forms were observed in the cytosol, suggesting that they are not deglycosylated by Png1 (Figure S1).

In conclusion, we show here that mutations in RTA can lead to structural modifications that target RTA to distinct trafficking pathways. While some mutations prevent vacuole targeting and target preRTA only to the ERAD pathway for degradation, others are targeted to the vacuole as wild type preRTA, but differ from wild type in their requirements for Png1. Wild type RTA has a distinct requirement for Png1 compared to the G83D variant, suggesting that it uses a different mechanism to exit the ER. We propose that deglycosylation of wild type preRTA by Png1 in the cytosol may be a strategy to avoid degradation by the ERAD pathway to reach ribosomes. Other ER-targeted toxins, such as cholera toxin and Shiga toxins, as well as retroviral proteins, which exploit the ERAD pathway to enter the cytosol [9] may use a similar mechanism to avoid degradation by the ubiquitin proteasome machinery to reach their cytosolic targets.

## Materials and Methods

### Plasmids and yeast strains

Wild type preRTA contains a 35-residue N-terminal leader, followed by the 267-residue RTA (preRTA-EGFP, NT1205) and wild type mature RTA consists of the 267-residue RTA (matRTA-EGFP, NT1206). The precursor and mature forms of G83D, S215F, G212E and P95L/E145K were fused with EGFP at their 3′ end and cloned into the yeast vector containing the *LEU2* marker (NT198) downstream of the galactose-inducible *GAL1* promoter to generate preG83D-EGFP, NT1248; matG83D, NT1254; preS215F-EGFP, NT1251; matS215F-EGFP, NT1257; preG212E-EGFP, NT1250; matG212E-EGFP, NT1256; preP95L/E145K-EGFP, NT1252; matP95L/E145K-EGFP, NT1258). The cDNAs corresponding to precursor forms of RTA and RTA mutants were cloned into NT198 downstream of the *GAL1* promoter without the EGFP tag to generate preRTA, NT849; preG83D, NT1031; preS215F, NT1038; preG212E, NT1037; preP95L/E145K, NT1039) [21]. The plasmids were transformed into the *Saccharomyces cerevisiae* strain W303 (*MATa ade2-1 trp1-1 ura3-1 leu2-3,112 his3-11,15 can1-100*), and transformants were selected on SD-Leu medium containing 2% glucose. The yeast deletion strain *png1*Δ and the parental BY4743 (*MATa/a his3D1/his3D1 leu2D0/leu2D0 lys2D0/LYS2 MET15/met15D0 ura3D0/ura3D0*) were obtained from yeast genome homozygous diploid gene deletion collection (Open Biosystems, Huntsville, AL). To investigate the effect of Png1 on preRTA, preG83D, preG212E, preS215F and preP95L/E145K, NT849, NT1031, NT1037, NT1038 and NT1039 were transformed into *png1*Δ, as well as the parental BY4743. The transformants were selected on SD-Leu medium containing 2% glucose. For complementation experiment, PNG1 ORF (YPL096W) was obtained from the yeast ORF library (Open Biosystems, Huntsville, AL) [43]. The PNG1 ORF was cloned into pAG415GPD-ccdB-HA (Addgene plasmid 14242) [44] with the *LEU2* marker using the Gateway cloning system (Invitrogen, Eugene, Oregon). The preRTA and preG83D plasmids were cloned into the single copy CEN plasmid, pRS416 [45] with *URA3* marker (preRTA, NT1403; preG83D, NT1541).

### Analysis of protein expression

Yeast cells were grown in dropout medium supplemented with 2% glucose overnight and then transferred to dropout medium supplemented with 2% galactose at OD_600_ of 0.3 to induce RTA expression. Cells were collected at 6 hpi and membrane fractions were isolated as previously described [46]. The supernatant was further centrifuged at 200,000×g for 1 h to pellet the ribosomes. The membrane fraction and the post ribosomal supernatant were treated with or without Endo H using the manufacturer's protocol (New England Biolabs, Ipswich, MA). The protein samples were separated on a 10% SDS-polyacrylamide gel and the blot was probed with monoclonal anti-RTA (1∶5000), a gift of Dr. Nicholas J. Mantis. The blot was stripped with 8 M guanidine hydrochloride and reprobed with antibody against the ER marker, dolichol phosphate mannose synthase (Dpm1p; Invitrogen, Eugene, Oregon) (1∶1000). The blot was also reprobed using anti-3-phosphoglycerate kinase (Pgk1p; Invitrogen, Eugene, Oregon), as a marker for the cytosol. The blots were developed using ChemiDoc MP imaging system (Bio-Rad, Philadelphia, PA). The vacuole fraction was isolated as described [47]. Briefly, cells harvested at 6 hpi were washed with DTT solution (0.1 M Tris pH9.4, 10 mM DTT) and lysed by lyticase at 20 U/OD in spheroplasting buffer (0.16× YPD, 0.4 M sorbitol, 50 mM potassium phosphate, pH 7.5). The spheroplasts were treated with DEAE-Dextran for gentle lysis. Purified vacuoles were obtained by flotation in a 0, 4, 8, 15% Ficoll step gradient. The gradients were centrifuged at 110,000×g for 90 min at 4°C in a Beckman L8-70 M Ultracentrifuge (Beckman Coulter, Brea, CA) and vacuoles were collected from the 0%–4% interface. The vacuole marker was anti-H^+^-ATPase (V-ATPase; Invitrogen, Eugene, Oregon) (1∶500). The total protein extracts were isolated as described [48]. Briefly, the 5 OD_600_ cells were washed with water. The cells were resuspended in 2 M LiAc and kept on ice for 5 min, followed by treating with 0.4 M NaOH and incubated on ice for 5 min.

### Cell viability analysis

Yeast cells carrying precursor and mature forms of RTA and nontoxic RTA mutants with the EGFP tag were induced as described above. Tenfold serial dilutions of 15 µL from OD_600_ of 10^−1^ to 10^−5^ were plated on SD-Leu plates containing 2% glucose at 10 hpi. BY4743 and *png1*Δ strains expressing preRTA or co-expressing pAG415GPD-PNG or vector and pRS416Gal1-preRTA were induced as described above. A series of 10-fold dilutions of 10 µL from OD_600_ of 10^−1^ to 10^−5^ were plated on dropout plates containing 2% glucose at 0 and 24 hpi. The plates were incubated at 30°C for approximately 48 h. BY4743 and *png1*Δ strains expressing preG83D or co-expressing pAG415GPD-PNG or vector and pRS416Gal1-preG83D were grown overnight in glucose as described above. The non-induced overnight culture were collected and plated on dropout plate with 2% galactose in a series of 10-fold dilutions of 10 µL from OD_600_ of 10^−1^ to 10^−5^. The plates were incubated at 30°C for 4 days. The CFU/ml was calculated based on the analysis of three different transformants at each time point.

### Depurination analysis

Total RNA was extracted using the RNeasy Mini Kit (Qiagen, Valencia, CA). For *in vitro* depurination, yeast ribosomes were isolated as previously described [49]. Ribosomes were incubated for 10 minutes at 30°C with purified wild type RTA at final concentrations of 10, 20, 40, 80, 100, 200, 400, 800, 1000 and 2000 pM or with G212E or P95L/E145K mutants at final concentrations of 1, 2, 4, 8, 10, 20, 40, 80 and 100 nM. Total RNA was isolated from ribosomes. The qRT-PCR analysis of depurination was carried out as previously described [24], [50]. Total RNA was converted to cDNA using the High Capacity cDNA Reverse Transcription Kit (Applied Biosystems, Carlsbad, CA). The 25S rRNA was detected using (5′-AGA CCG TCG CTT GCT ACA AT-3′and 5′- ATG ACG AGG CAT TTG GCT AC- 3′). The depurinated rRNA was detected using the forward primer (5′- CTA TCG ATC CTT TAG TCC CTC-3′) and the reverse primer (5′- CCG AAT GAA CTG TTC CAC A-3′). Real time PCR was performed using an ABI Prism 7000 Sequence Detection System (Applied Biosystems, Carlsbad, CA). Three replicates of each sample were analyzed by the comparative ΔC_T_ (ΔΔC_T_) method for quantification [24].

### Live cell imaging

Time course of RTA localization was carried out using yeast harboring the precursor and mature RTA with EGFP tag at indicated time points. The cells were directly added to 2% agarose pads on slides. For vacuole stain, yeast cells were harvested at 2, 4, 6 and 24 hpi. FM4-64 (Invitrogen, Carlsbad, CA) dissolved in dimethyl sulfoxide (DMSO) was added at a final concentration of 80 µM and cells were incubated in the dark at 30°C for 60 min. Cells were pelleted, washed and resuspended with YPD media containing 2% galactose to chase for 40 min at 30°C. To stain the nuclei, Hoechst 33342 (Invitrogen, Carlsbad, CA) was added at a final concentration of 10 µM to the cell culture and incubated at 37°C for 60 min. The cell culture was applied to the agar pad and visualized using an Olympus BX41 fluorescence microscope equipped with a CCD camera (Hamamatsu, Bridgewater, NJ) and a 100× oil objective (1.45 N.A. Plan Apo, Olympus). Image acquisition and processing were performed using Metamorph Image Software (7.0; MDS Analytical Technologies).

### Strain verification

The *png1*Δ strain was verified by PCR using knockout cassette specific primers (primers “A” and “KanB”) (yeastdeletion.stanford.edu). The PCR was also conducted with the same primer pair for the parental strain BY4743 as a control.

### Statistical analysis

Comparison of two-sample treatment means was performed using independent two-sided Student's t-Tests with Origin 9.1 (OriginLab) software.

## Supporting Information

Figure S1 **The RTA variants preG212E and preP95L/E145K are not substrates for Png1.** (A) The viability of BY4743 and *png1*Δ expressing preG212E and preP95L/E145K. A series of ten-fold dilutions were spotted on glucose and galactose plates after overnight growth in glucose. (B) Immunoblot analysis of BY4743 and *png1*Δ expressing preG212E and preP95L/E145K. The membrane fraction (M) and cytosol fraction (C) isolated at 6 and 24 hpi were separated on a 10% SDS-polyacrylamide gel and probed with monoclonal anti-RTA (1∶5000). The ER membrane marker Dpm1p and cytosolic marker Pgk1p were used as loading controls. (C) Ribosome depurination by wild type preRTA, preG212E and preP95L/E145K expressed in BY4743 and *png1*Δ by qRT-PCR at 4 hpi.
(PDF)

Figure S2 **The RTA variant preS215F is not a substrate for Png1.** (A) The viability of BY4743 and *png1*Δ expressing preS215F. A series of 10-fold dilutions were spotted on glucose and galactose plates after overnight growth in glucose. (B) Immunoblot analysis of BY4743 and *png1*Δ expressing preS215F. The membrane fraction (M) and cytosol fraction (C) isolated at 6 and 24 hpi were separated on a 10% SDS-polyacrylamide gel and probed with monoclonal anti-RTA (1∶5000). The ER membrane marker Dpm1p and cytosolic marker Pgk1p were used as loading controls. (C) Ribosome depurination by preS215F expressed in BY4743 and *png1*Δ transformed with preS215F by qRT-PCR at 2, 4, and 6 hpi.
(PDF)

Figure S3 **The nonglycosylated RTA mutant is not a substrate for Png1.** (A) The viability of BY4743 and *png1*Δ expressing the nonglycosylated RTA mutant preN10Q/N236Q. A series of ten-fold dilutions were spotted on a glucose plate at 0 and 24 h post induction. (B) Ribosome depurination by preN10Q/N236Q in BY4743 and *png1*Δ was analyzed by qRT-PCR at 2 and 4 hpi.
(PDF)

Figure S4 **Expression of Png1 in** ***png1Δ*** **expressing preRTA.** (A) Analysis of viability of *png1*Δ and BY4743 co-expressing preRTA and *PNG1* driven by the constitutive *GPD1* promoter. A series of 10-fold dilutions were spotted on glucose plate at 0 and 24 h post induction in galactose media. The CFU/ml was calculated based on the analysis of at least three different transformants. (B) Immunoblot analysis of *png1*Δ and BY4743 co-expressing preRTA and *PNG1* or harboring the *PNG1* vector. Total protein isolated at 6 hpi was separated on a 10% SDS-polyacrylamide gel and probed with monoclonal anti-RTA (1∶5000). The blot was reprobed with anti-HA (1∶1000) to detect the expression of C-terminal HA tagged Png1. The ER membrane marker Dpm1p and cytosolic marker Pgk1p were used as loading controls. (C) Ribosome depurination by preRTA in *png1*Δ and BY4743 transformed with *PNG1* was analyzed by qRT-PCR at 2, 4, and 6 hpi.
(PDF)

Figure S5 **Expression of Png1 in** ***png1Δ*** **expressing preG83D.** (A) Analysis of viability *png1*Δ and BY4743 co-expressing preG83D and *PNG1* driven by the constitutive *GPD1* promoter. A series of 10-fold dilutions were spotted on a galactose plate after overnight growth in glucose media. The CFU/ml was calculated based on the analysis of at least three different transformants. (B) Immunoblot analysis of *png1*Δ and BY4743 co-expressing preG83D and *PNG1* or harboring the *PNG1* vector. Total protein isolated at 6 hpi was separated on a 10% SDS-polyacrylamide gel and probed with monoclonal anti-RTA (1∶5000). The blot was reprobed with anti-HA (1∶1000) to detect the expression of C-terminal HA tagged Png1. The ER membrane marker Dpm1p and cytosolic marker Pgk1p were used as loading controls. (C) Ribosome depurination by preG83D in *png1*Δ or in BY4743 transformed with *PNG1* was analyzed by qRT-PCR at 2, 4, and 6 hpi.
(PDF)
