# Supplementary material for: Wild Type RTA and Less Toxic Variants Have Distinct Requirements for Png1 for Their Depurination Activity and Toxicity in Saccharomyces cerevisiae
Source: PLoS One. 2014 Dec 1;9(12):e113719. doi: 10.1371/journal.pone.0113719 (PMC4250064; doi:10.1371/journal.pone.0113719)
Supplement: Figure S1 — The RTA variants preG212E and preP95L/E145K are not substrates for Png1. (A) The viability of BY4743 and png1Δ expressing preG212E and preP95L/E145K. A series of ten-fold dilutions were spotted on glucose and galactose plates after overnight growth in glucose. (B) Immunoblot analysis of BY4743 and png1Δ expressing preG212E and preP95L/E145K. The membrane fraction (M) and cytosol fraction (C) isolated at 6 and 24 hpi were separated on a 10% SDS-polyacrylamide gel and probed with monoclonal anti-RTA (1∶5000). The ER membrane marker Dpm1p and cytosolic marker Pgk1p were used as loading controls. (C) Ribosome depurination by wild type preRTA, preG212E and preP95L/E145K expressed in BY4743 and png1Δ by qRT-PCR at 4 hpi. (PDF) [file pone.0113719.s001.pdf]

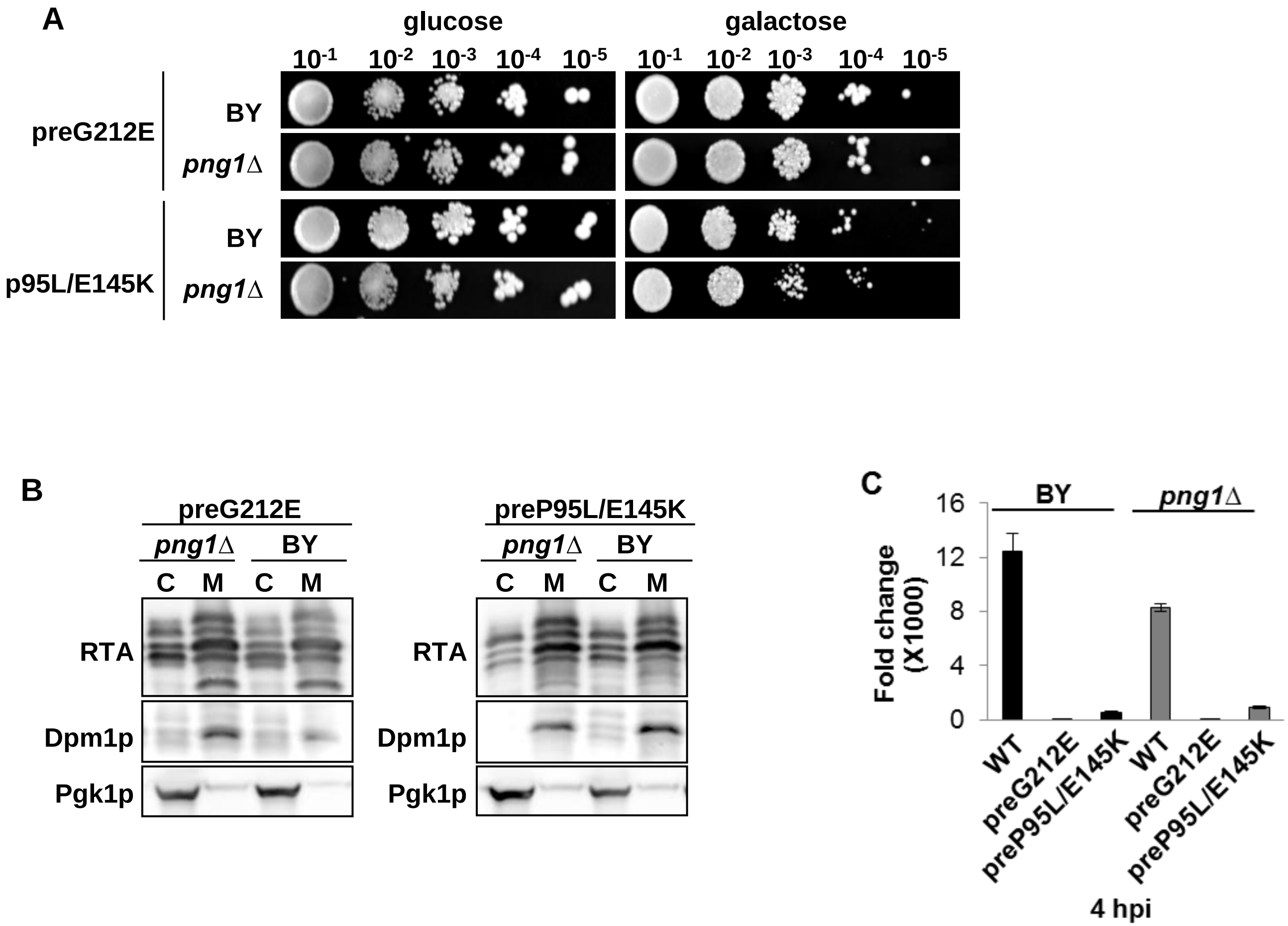

Figure S1. The RTA variants preG212E and preP95L/E145K are not substrates for Png1. (A) The viability of BY4743 and *png1*Δ expressing preG212E and preP95L/E145K. A series of ten-fold dilutions were spotted on glucose and galactose plates after overnight growth in glucose. (B) Immunoblot analysis of BY4743 and *png1*Δ expressing preG212E and preP95L/E145K. The membrane fraction (M) and cytosol fraction (C) isolated at 6 and 24 hpi were separated on a 10% SDS-polyacrylamide gel and probed with monoclonal anti-RTA (1:5000). The ER membrane marker Dpm1p and cytosolic marker Pgk1p were used as loading controls. (C) Ribosome depurination by wild type preRTA, preG212E and preP95L/E145K expressed in BY4743 and *png1*Δ by qRT-PCR at 4 hpi.
