# Supplementary material for: Wild Type RTA and Less Toxic Variants Have Distinct Requirements for Png1 for Their Depurination Activity and Toxicity in Saccharomyces cerevisiae
Source: PLoS One. 2014 Dec 1;9(12):e113719. doi: 10.1371/journal.pone.0113719 (PMC4250064; doi:10.1371/journal.pone.0113719)
Supplement: Figure S2 — The RTA variant preS215F is not a substrate for Png1. (A) The viability of BY4743 and png1Δ expressing preS215F. A series of 10-fold dilutions were spotted on glucose and galactose plates after overnight growth in glucose. (B) Immunoblot analysis of BY4743 and png1Δ expressing preS215F. The membrane fraction (M) and cytosol fraction (C) isolated at 6 and 24 hpi were separated on a 10% SDS-polyacrylamide gel and probed with monoclonal anti-RTA (1∶5000). The ER membrane marker Dpm1p and cytosolic marker Pgk1p were used as loading controls. (C) Ribosome depurination by preS215F expressed in BY4743 and png1Δ transformed with preS215F by qRT-PCR at 2, 4, and 6 hpi. (PDF) [file pone.0113719.s002.pdf]

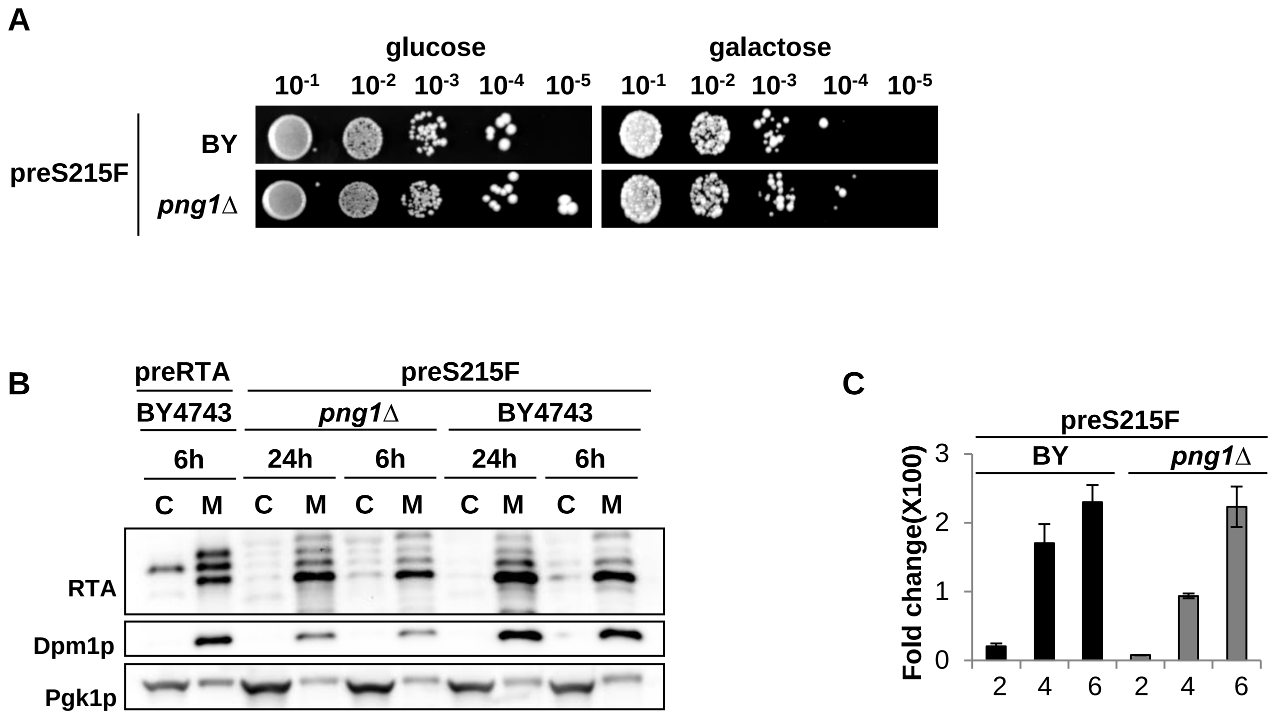

Figure S2. The RTA variant preS215F is not a substrate for Png1. (A) The viability of BY4743 and *png1*Δ expressing preS215F. A series of 10-fold dilutions were spotted on glucose and galactose plates after overnight growth in glucose. (B) Immunoblot analysis of BY4743 and *png1*Δ expressing preS215F. The membrane fraction (M) and cytosol fraction (C) isolated at 6 and 24 hpi were separated on a 10% SDS-polyacrylamide gel and probed with monoclonal anti-RTA (1:5000). The ER membrane marker Dpm1p and cytosolic marker Pgk1p were used as loading controls. (C) Ribosome depurination by preS215F expressed in BY4743 and *png1*Δ transformed with preS215F by qRT-PCR at 2, 4, and 6 hpi.
