# Supplementary material for: Wild Type RTA and Less Toxic Variants Have Distinct Requirements for Png1 for Their Depurination Activity and Toxicity in Saccharomyces cerevisiae
Source: PLoS One. 2014 Dec 1;9(12):e113719. doi: 10.1371/journal.pone.0113719 (PMC4250064; doi:10.1371/journal.pone.0113719)
Supplement: Figure S3 — The nonglycosylated RTA mutant is not a substrate for Png1. (A) The viability of BY4743 and png1Δ expressing the nonglycosylated RTA mutant preN10Q/N236Q. A series of ten-fold dilutions were spotted on a glucose plate at 0 and 24 h post induction. (B) Ribosome depurination by preN10Q/N236Q in BY4743 and png1Δ was analyzed by qRT-PCR at 2 and 4 hpi. (PDF) [file pone.0113719.s003.pdf]

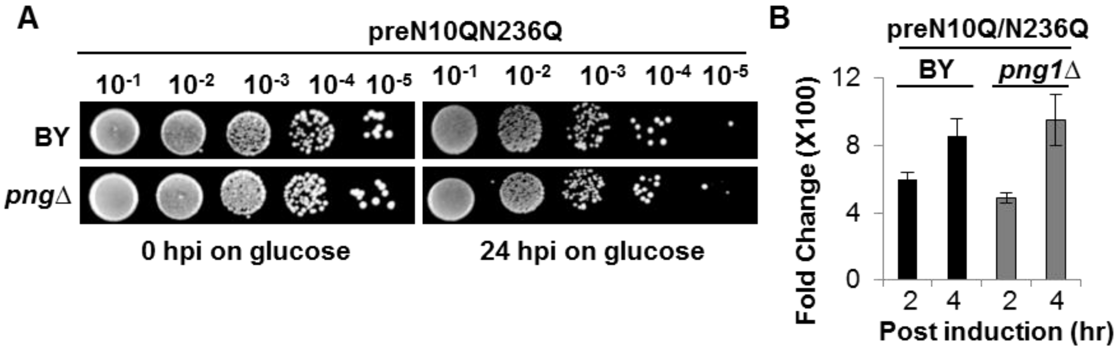

Figure S3. The nonglycosylated RTA mutant is not a substrate for Png1. (A) The viability of BY4743 and *png1Δ* expressing the nonglycosylated RTA mutant preN10Q/N236Q. A series of ten-fold dilutions were spotted on a glucose plate at 0 and 24 h post induction. (B) Ribosome depurination by preN10Q/N236Q in BY4743 and *png1Δ* was analyzed by qRT-PCR at 2 and 4hpi.
