# Supplementary material for: Wild Type RTA and Less Toxic Variants Have Distinct Requirements for Png1 for Their Depurination Activity and Toxicity in Saccharomyces cerevisiae
Source: PLoS One. 2014 Dec 1;9(12):e113719. doi: 10.1371/journal.pone.0113719 (PMC4250064; doi:10.1371/journal.pone.0113719)
Supplement: Figure S5 — Expression of Png1 in png1Δ expressing preG83D. (A) Analysis of viability png1Δ and BY4743 co-expressing preG83D and PNG1 driven by the constitutive GPD1 promoter. A series of 10-fold dilutions were spotted on a galactose plate after overnight growth in glucose media. The CFU/ml was calculated based on the analysis of at least three different transformants. (B) Immunoblot analysis of png1Δ and BY4743 co-expressing preG83D and PNG1 or harboring the PNG1 vector. Total protein isolated at 6 hpi was separated on a 10% SDS-polyacrylamide gel and probed with monoclonal anti-RTA (1∶5000). The blot was reprobed with anti-HA (1∶1000) to detect the expression of C-terminal HA tagged Png1. The ER membrane marker Dpm1p and cytosolic marker Pgk1p were used as loading controls. (C) Ribosome depurination by preG83D in png1Δ or in BY4743 transformed with PNG1 was analyzed by qRT-PCR at 2, 4, and 6 hpi. (PDF) [file pone.0113719.s005.pdf]

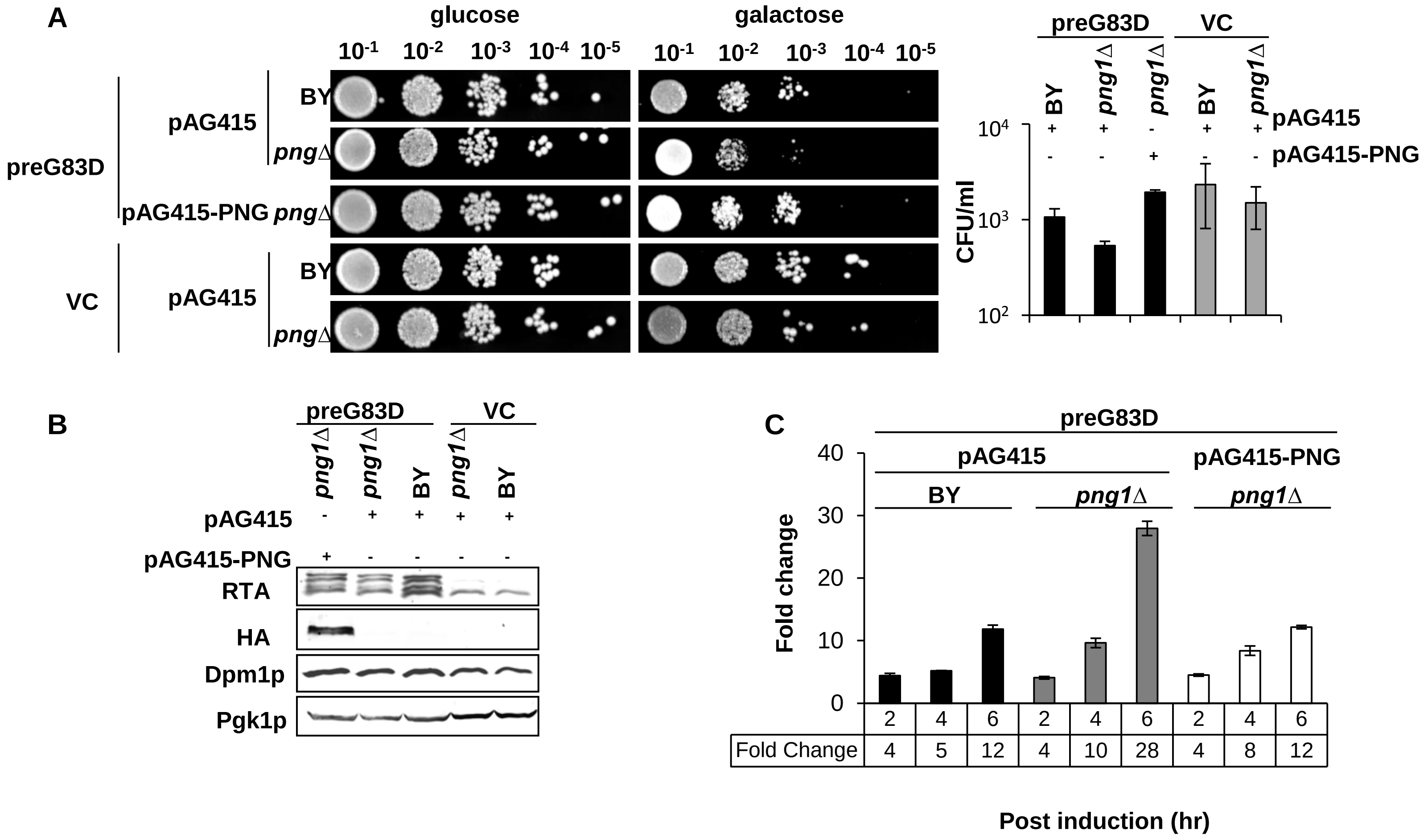

Figure S5. Expression of Png1 in *png1*Δ expressing preG83D. (A) Analysis of viability *png1*Δ and BY4743 co-expressing preG83D and *PNG1* driven by the constitutive *GPD1* promoter. A series of 10-fold dilutions were spotted on a galactose plate after overnight growth in glucose media. The CFU/ml was calculated based on the analysis of at least three different transformants. (B) Immunoblot analysis of *png1*Δ and BY4743 co-expressing preG83D and *PNG1* or harboring the *PNG1* vector. Total protein isolated at 6 hpi was separated on a 10% SDS-polyacrylamide gel and probed with monoclonal anti-RTA (1:5000). The blot was reprobed with anti-HA (1:1000) to detect the expression of C-terminal HA tagged Png1. The ER membrane marker Dpm1p and cytosolic marker Pgk1p were used as loading controls. (C) Ribosome depurination by preG83D in *png1*Δ or in BY4743 transformed with *PNG1* was analyzed by qRT-PCR at 2, 4 and 6 hpi.
